# Precision Assessment of Facial Asymmetry Using 3D Imaging and Artificial Intelligence

**DOI:** 10.3390/jcm14207172

**Published:** 2025-10-11

**Authors:** Mohamed Adel, Katie Jo Hunt, Daniel Lau, James K. Hartsfield, Hugo Reyes-Centeno, Cynthia S. Beeman, Tarek Elshebiny, Lina Sharab

**Affiliations:** 1 Department of Orthodontics, College of Dentistry, Texas A&M University, 3302 Gaston Avenue, Room 719, Dallas, TX 75246, USA; 2 Private Practice, 5 Overlook Dr #6, Amherst, NH 03031, USA; kjhunt01@gmail.com; 3 Department of Electrical and Computer Engineering, Pigman College of Engineering, University of Kentucky, Lexington, KY 40506, USA; dllau@uky.edu; 4 Division of Orthodontics, College of Dentistry, University of Kentucky, Lexington, KY 40508, USA; james.hartsfield@uky.edu (J.K.H.); cbeeman@uky.edu (C.S.B.); lina.sharab@uky.edu (L.S.); 5 Department of Anthropology, College of Arts and Sciences, University of Kentucky, Lexington, KY 40508, USA; hugo.reyes-centeno@uky.edu; 6 Swedish Collegium for Advanced Study, Linneanum, Villavägen 6c, SE-752 36 Uppsala, Sweden; 7 Center for the Human Past-Uppsala & Stockholm Universities, Evolutionary Biology Centre, Norbyvägen 18A, 752 36 Uppsala, Sweden; 8 Department of Orthodontics, School of Dental Medicine, Case Western Reserve University, Cleveland, OH 44106, USA; tme18@case.edu

**Keywords:** artificial intelligence, deep learning, agreement, 3D facial images, Vectra M3, stereophotogrammetry, facial asymmetry, landmark detection

## Abstract

**Objectives**: There is a growing interest among practitioners in employing artificial intelligence (AI) to enhance the precision and efficiency of diagnostic methods. The objective of this study is to assess the precision of an AI-based method for facial asymmetry assessment using 3D facial images. **Methods**: The study included 130 patients (84 female, 46 male), analyzing 3D facial images from the Vectra^®^ M3 imaging system using both manual and AI-based methods. Seven bilateral facial landmarks were identified for manual analysis, calculating the asymmetry index for facial symmetry assessment. An AI-based program was developed to automate the identification of the same landmarks and calculate the asymmetry index. The reliability of the manual measurements was assessed using intraclass correlation coefficients (ICC) with 95% confidence intervals (CI). Precision of automated landmark identification was compared to the manual method. **Results**: The ICCs for the manual measurements demonstrated moderate to excellent reliability, both within raters (ICC = 0.62–0.99) and between raters (ICC = 0.72–0.96) each calculated with 95% CI. Agreement was observed between the manual and automated methods in calculating the asymmetry index for five landmarks. There was a statistically significant difference between the two methods in determining the asymmetry index for alare (median: 2.05 mm manual vs. 1.54 mm automated, *p* = 0.0056) and cheilion (median: 2.77 mm manual vs. 2.30 mm automated, *p* = 0.0081). **Conclusions**: The AI-based method provides efficient and comparable precision of facial asymmetry analysis using 3D images. The disagreement observed between the two methods can be addressed through further improvement and training of the automated software. This innovative approach opens doors to significant advancements in both research and clinical orthodontics.

## 1. Introduction

While the human face tends to develop with the right and left sides as mirror images of each other over the midline [1], several biological factors and environmental disturbances influence the facial development make perfect bilateral symmetry rare [2,3]. Differences in the relationship or size of the facial halves are defined as facial asymmetry [4]. Slight non-pathologic asymmetries, also known as subclinical asymmetries, are common anthropometric features of the human face that are almost indiscernible and do not require any treatment [2,3,5]. A more severe degree of facial asymmetry with perceptible variations between the two halves of the face is considered clinically significant since it may indicate pathologies of the broader craniofacial region. Therefore, quantifying facial asymmetry is crucial to differentiate between patients with subclinical asymmetry and those needing complex orthodontic or surgical intervention [6]. 

Patients with facial asymmetry are evaluated through direct anthropometry, clinical examination, facial photos, and radiographic examination [7]. Until recently, two-dimensional (2D) analysis techniques were commonly used to diagnose it, involving clinical facial photographs and cephalometric radiography in lateral and posteroanterior (PA) views [6]. However, properly evaluating 3D structures in 2D is a challenge. The introduction of 3D imaging techniques addressed this challenge and significantly broadened the scope of diagnosis and treatment planning in dentistry. The development of 3D radiographic imaging, such as computed tomography (CT) and cone beam computed tomography (CBCT), opened a new era in the diagnosis of facial asymmetry [8,9]. For example, a previous study [9] compared PA cephalograms, CBCTs, and physical caliper measurements (considered the gold standard) to evaluate facial asymmetry using ten dry human skulls. The results revealed poor reproducibility of reference points between the true physical measurements and the PA cephalograms and, by contrast, an almost perfect agreement when the true physical measurements were compared to the CBCTs. This suggests that measurements derived from CBCT imaging can be a superior tool for evaluating facial asymmetry [9]. However, concerns about radiation safety preclude its wide application [10]. A practical alternative, therefore, is applying a non-invasive 3D surface imaging technique, such as 3D stereophotogrammetry and laser scanning, for similarly conducting an extensive range of quantitative facial measurements. Previous studies have confirmed that accurate and reproducible identification of bone-related soft tissue landmarks can be achieved with 3D facial images, enabling a more precise assessment of craniofacial deformities [11,12,13]. Moreover, facial asymmetry can involve both vertical or transverse components, or a combination of both. Therefore, evaluating this asymmetry using 3D images would likely provide a more accurate assessment compared to 2D images [11,14].

In addition to the adoption of new data acquisition technology over the last decades, interest in using artificial intelligence has increased as orthodontics practitioners search for more accurate and efficient diagnostic modalities [15]. Artificial intelligence broadly refers to a system’s ability to mimic human-like intelligence and make correct and effective decisions [16]. One of the main subcategories of artificial intelligence is machine learning, a technique to provide predictions of new data and conditions based on the previously learned data’s statistical pattern. For instance, in the context of medical diagnoses, machines undergo training to discern and categorize diverse signs and symptoms, enabling them to generate likely diagnoses [17]. Another subtype of artificial intelligence is the artificial neural network, a computing system inspired by the biological neural networks that constitute brains [18]. It operates using interconnected data nodes (“neurons”) arranged in layered structures reminiscent of the human brain’s organization [19]. With rapid advancement in computational technology, scientists have developed increasingly complex and “deeper” neural networks to solve more intricate practical problems. This evolution has led to the recognition of these advanced neural networks as “deep learning” [20]. Deep learning does not require expensive engineering effort to preprocess the data and has been used in visual object recognition and object detection [21,22].

In orthodontics, diagnostic imaging is the most notable application for using deep learning. For instance, recent studies applying deep learning have revealed outstanding achievements in automatic landmark detection of lateral cephalograms [23,24], in automatically determining the stage of cervical vertebrae maturation [25,26], in classifying skeletal malocclusions [27] and in calculating pharyngeal airway volume [15,28]. Numerous studies have also applied deep learning methods to digital orthodontic photographs, specifically focusing on 2D images [29]. Rousseau and Retrouvey used 2D facial images to compare the facial proportions calculated from a manual image annotation to an automated facial vertical dimensions analysis program. They used pre-trained deep learning algorithms on identical facial landmarks [30]. The results of this study confirmed the efficacy of deep learning models as an automated alternative to manual measurement of photos to determine the patient’s facial vertical dimension. Recent studies have notably shifted focus towards utilizing deep learning and other artificial intelligence techniques on 3D facial images due to their capacity to provide a more precise representation of facial structures compared to traditional 2D images [11,12]. These investigations have primarily concentrated on landmark identification, facial recognition, and expression detection, showcasing the effectiveness of artificial intelligence in these specific functions [31,32]. Considering the success achieved thus far, it is logical to extend these efforts towards comprehensive facial analysis.

To date, no reports in the literature have evaluated automated methodologies employing artificial intelligence for identifying patients with facial asymmetry using 3D facial images. In this study, we focus on facial symmetry—a feature of clinical significance that benefits from evaluation in 3D. The objective of this study is to validate an artificial intelligence-based method for facial asymmetry assessment, comparing its performance with a manual approach. Specifically, we apply a deep multi-view learning model (MVLM) [32] to identify landmarks on patients’ 3D facial surface images and use these to generate a facial asymmetry index automatically. A previous study concluded that facial asymmetry of less than 3 mm (or <3% right–left difference) is generally undetectable in a normal face [33]. Given previous success in applying deep MVLM, we expect that the null hypothesis will be supported, i.e., that facial asymmetry index scores between the deep MVLM and manual landmark identification approaches will demonstrate clinically acceptable agreement, thus validating the deep MVLM. This endeavor could provide a valuable tool for enhancing accuracy in diagnosis and treatment planning for individuals affected by facial asymmetry.

## 2. Materials and Methods

### 2.1. Sample

This is a cross-sectional study that used pretreatment facial scans of consecutively enrolled patients from the Orthodontic Graduate Clinic at the University of Kentucky- College of Dentistry. No restrictions were applied regarding sex, age or ethnicity. Inclusion criteria required patients to be actively receiving or having completed orthodontic treatment with complete diagnostic records. An analysis was performed using G*Power [34] (version 3.1.9.6) to determine sample size parameters. Using a two-tail design, the effect size of 0.5 and a total sample size of 126 produced an estimated *α* error probability of 0.00001 and power of 0.80. The effect size of 0.5 was selected to explore if the difference between manual and automated methods was moderate or greater. We initially recruited 130 patients (46 males, 84 females), anticipating the possibility of excluding some patients during the analysis phase. The age range of the sample is between 15 and 45 years (median 18, 25th percentile (Q_1_) = 16, 75th percentile (Q_3_) = 21).

### 2.2. Three-Dimensional Facial Surface Imaging

**Figure 1:**
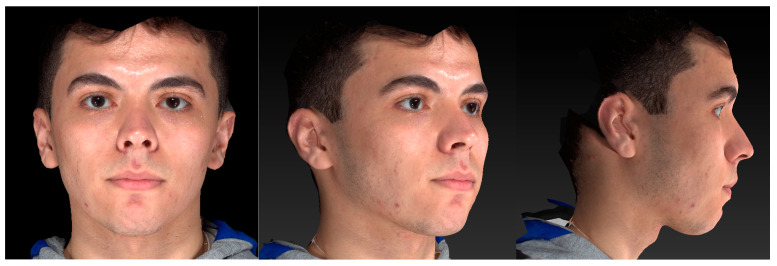
A 3D facial surface image obtained by the Vectra^®^ M3 imaging system showing frontal, three-quarter, and profile views.

This study utilized 3D facial photographs obtained by the Vectra^®^ M3 imaging system (Canfield Scientific, Fairfield, NJ, USA), comprising a 3D stereophotogrammetric camera setup for each patient. The system has been previously validated for clinical applications [35] and applied in the context of the study of facial asymmetry [36]. The camera setup comprises three pods, each equipped with two digital cameras and a light flash, with the center pod featuring a small mirror. All the photographs were taken with the participants in repose, maintaining a neutral facial expression and eyes open. The images were captured in the natural head position, with patients being asked to swallow and maintain occlusion of their molars while observing themselves in the mirror to achieve habitual occlusion. Participants were required to remove their glasses, pull their hair away from their faces, sit and stay still during the photo acquisition. Additionally, participants were instructed to remove makeup, and individuals with dense facial hair were excluded. Following photographic image capture, achieved within milliseconds, the software program VECTRA^®^ 3D Analysis Module (VAM version 6.2.3) was used to process a composite 3D surface image. The criteria for acceptable 3D facial surface images included valid representation of facial morphology, high scan resolution, absence of noticeable motion artifacts, and a closed mouth unless lips were naturally incompetent. To ensure measurement accuracy, the Vectra^®^ M3 imaging system was calibrated on a monthly basis, following the manufacturer’s recommended calibration procedures. Figure 1 shows that the system can faithfully replicate the facial surface geometry while accurately applying lifelike color and texture data to the geometric shape, resulting in a realistic rendering.

### 2.3. Measurements

#### 2.3.1. Manual Analysis

##### Head Orientation

**Figure 2:**
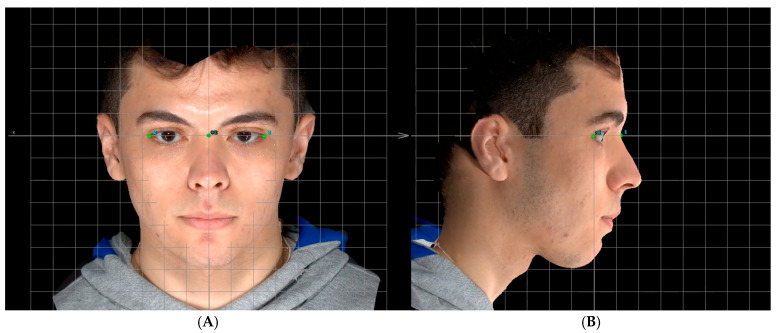
The process of head orientation. (**A**) Frontal view: Orienting the transverse plane (*XY* plane) connecting the nasion (1) and bilateral exocanthion (2 and 3) and the midfacial (sagittal) plane (*YZ*) perpendicular to it. (**B**) Profile view: Orienting the transverse plane (*XY* plane) and coronal plane (*XZ*) perpendicular to it.

The head orientation of the 3D surface was standardized within the reference framework using the VAM software with the agreement of the two observers (MA and KJH) at the same setting. The resulting orientation was then used by both observers for all subsequent landmarking steps. The process involved retrieving the image by the software and projecting the *X*, *Y*, and *Z* reference planes. First, the frontal view was viewed, and the head was adjusted until the midfacial (sagittal) plane (*YZ*) passed through nasion and was perpendicular to the transverse plane (*XY* plane) connecting the nasion and bilateral exocanthion (Figure 2A). Then, the head was turned to the lateral profile view, and the intersection between the transverse plane (*XY* plane) and coronal plane (*XZ*) was aligned with the exocanthion points (Figure 2B) [37]. This step is essential for standardizing the 3D facial images across the three spatial planes, ensuring comparable *x*, *y*, and *z* coordinates, and evaluating the reproducibility of identifying facial landmarks within and between observers.

##### Manual Landmarks Identification

**Figure 3:**
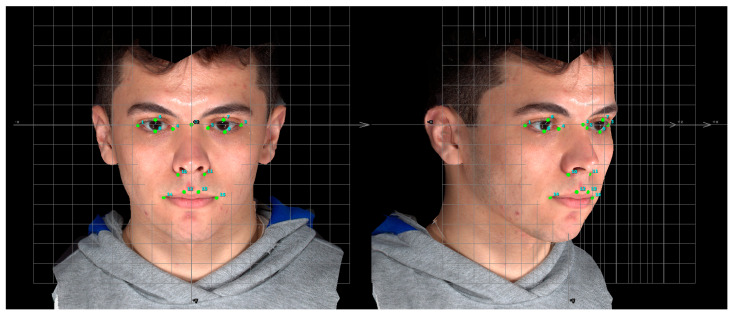
Facial landmarks were identified on the 3D facial images (see Table 1 for reference and definitions). Nasion (labeled 2).

Seven bilateral landmarks were identified manually and independently on each image by the two observers (MA and KJH) for the subsequent facial asymmetry analysis (Figure 3). Observers MA and KJH have 3 years of experience using the Vectra^®^ M3 imaging system. The bilateral landmarks are: palpebrale superius, palpebrale inferius, exocanthion, endocanthion, alare, crista philtra and cheilion (Figure 3, Table 1) [11,12]. The absolute values of the *x*, *y*, *z* coordinates of each of the seven bilateral landmarks were exported, in millimeters (mm), to a Microsoft Excel spreadsheet for subsequent analysis of facial asymmetry.

#### 2.3.2. Artificial Intelligence-Based Analysis

##### Model and Datasets

This study followed the Checklists for Artificial Intelligence in Medical Imaging (CLAIM) and Transparent Reporting of a multivariable prediction model for Individual Prognosis Or Diagnosis (TRIPOD) to ensure transparent and standardized reporting of methodology and results [38,39]. The 3D images of the sample were extracted in OBJ file format using the VAM software and processed using a computer programming script based on a deep MVLM for automated landmark identification [32]. The deep MVLM was developed using the Python 3.7 programming language and the PyTorch library 1.2 with training conducted on a Titan X GPU. The architecture consists of multiple convolutional and fully connected layers optimized for landmark detection, trained using cross-entropy loss with the Adam optimizer. The batch size was set at 32, with learning rate initialized at 0.001 and models trained for 200 epochs. Preprocessing included centering and aligning each 3D scan to the origin with the nose oriented along the z-axis, followed by normalization of the mesh geometry. Data augmentation during training included random rotations, scaling, and mirroring to improve robustness. This programming script utilized a pre-trained artificial intelligence model using data from two publicly available datasets: the Binghamton University 3D Facial Expression dataset (BU-3DFE) [40] and the Universiti Putra Malaysia Facial Expression Recognition Database (UPM-3DFE) [41]. The BU-3DFE dataset comprises 3D surface facial images of 100 subjects (56 females, 44 males), each marked with 83 landmarks. The UPM-3DFE database contains 3D facial images of 50 subjects (20 female, 30 male), each scan annotated with 32 landmarks. [32] Both datasets sample individuals from diverse ethnic backgrounds. The facial images on both datasets have been cropped to isolate only the facial region and then transformed into a 3D point cloud model. These datasets were used to train the deep MVLM to accurately orient the head and identify the specific landmarks for this study, as described below. While the datasets feature diverse emotional expressions of each individual, only the neutral expression (i.e., in repose) was used to train the model. The source code is available on GitHub under the MIT license, with full documentation in the accompanying README file (https://github.com/RasmusRPaulsen/Deep-MVLM, accessed on 3 October 2025).

##### Head Orientation

**Figure 4:**
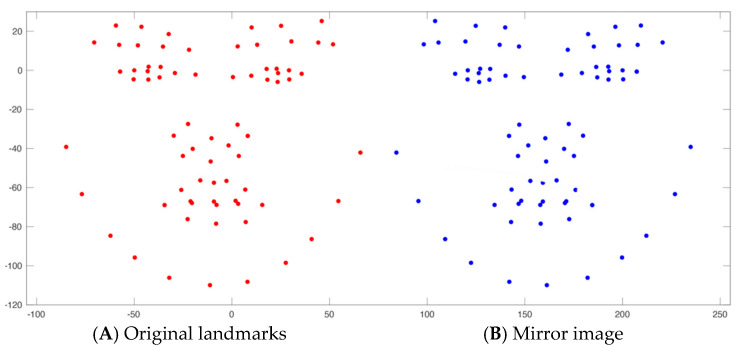
(**A**) Depicts the original facial landmarks (N = 115) projected onto the cloud model of the image. (**B**) The same facial landmarks were duplicated and mirrored along the Y-axis, creating mirror images for all landmarks.

**Figure 5:**
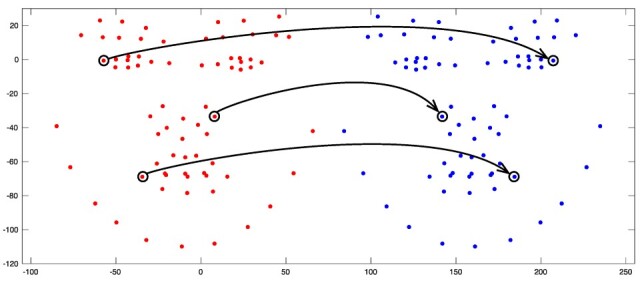
Representation of the landmark matching process during transformation.

**Figure 6:**
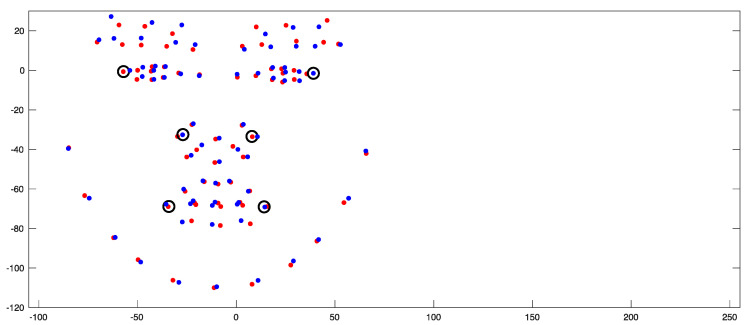
Alignment process between original (red) and mirror-imaged (blue) faces. The Black circles show some of the bilateral landmarks used in the alignment process.

**Figure 7:**
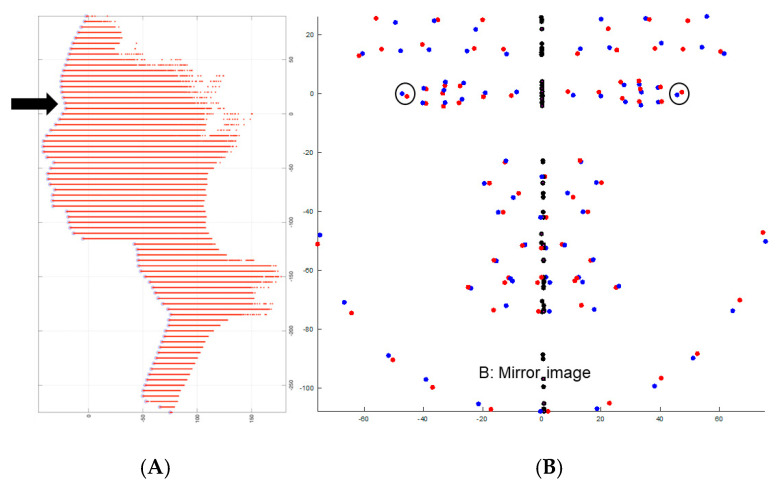
Identification of the midfacial plane (YZ). (**A**) Depicts the identification of nasion point. (**B**) Depicts the identification of the midfacial plane (YZ) passing through nasion and between the bilateral exocanthion.

**Figure 8:**
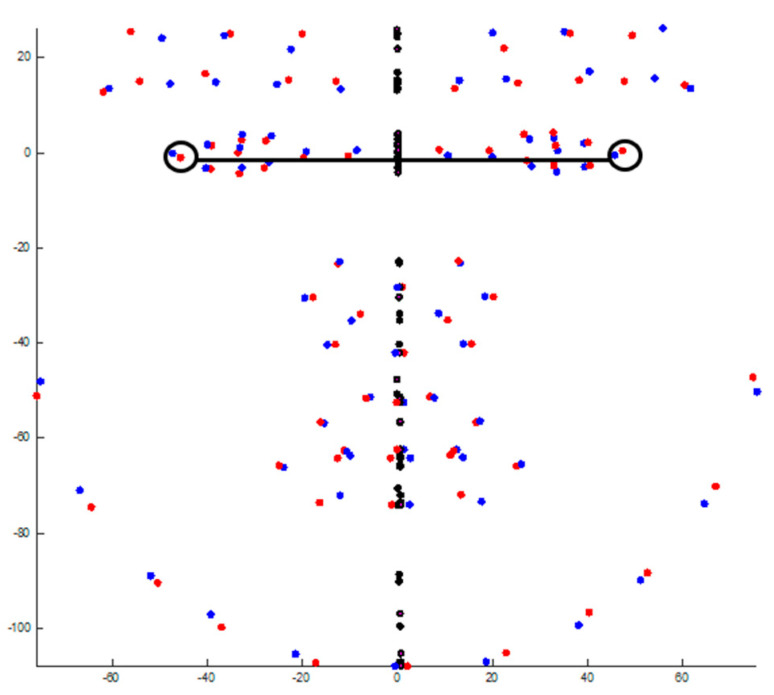
The midfacial (sagittal) plane (YZ) and transverse plane (XY) perpendicular to each other.

**Figure 9:**
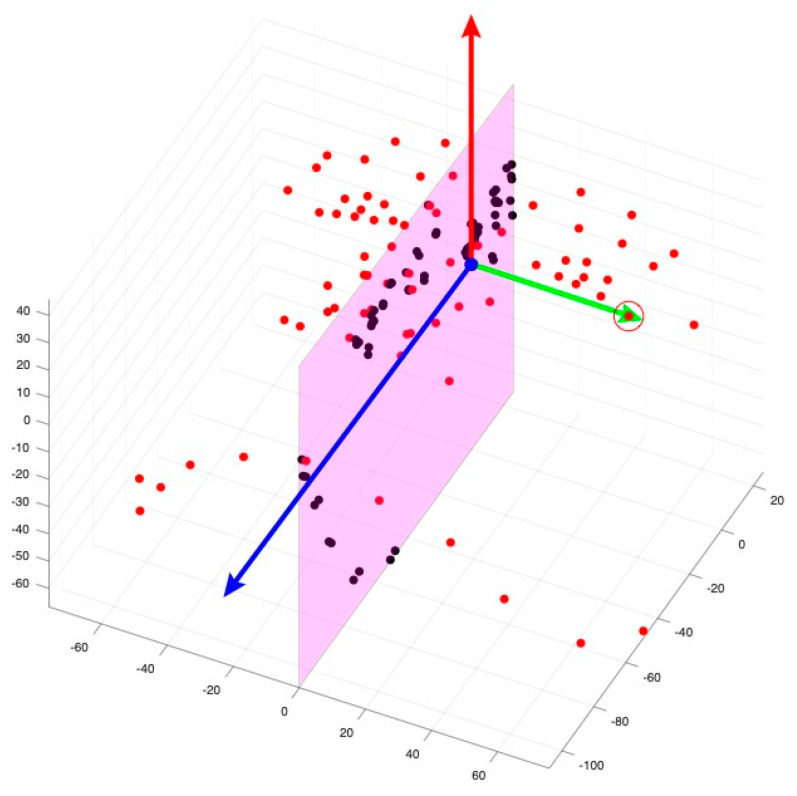
Determination of axes and planes in facial orientation. The red vector represents the negative direction of the YZ-axis. The green vector signifies the negative direction of the XY-axis. The blue vector illustrates the established coronal plane (XZ) derived from the cross product of the YZ and XY axes.

The head orientation process begins with the model projecting all the common landmarks (N = 115) from the two databases onto the cloud model of each image to identify facial features. The model failed to identify landmarks on the facial images of six subjects, likely due to distortion in the point cloud data. These subjects were excluded from the sample due to inability to complete landmark identification. As a result, these subjects were excluded from the analysis. These landmarks are duplicated and flipped along the Y-axis to create mirror images of all the facial features (Figure 4). Each original landmark is then matched with its counterpart on the other side of the mirror image to find the best transformation for the face (Figure 5). The original face is then aligned with its mirror-imaged version by pairing the point clouds of both faces using the iterative closest point (Figure 6). The subsequent step involves determining the midfacial (sagittal) plane (YZ), which is identified by locating the midpoint between bilateral exocanthions and passing through nasion (Figure 7) in the same fashion as in the manual approach. Next, the nasion and bilateral exocanthions are connected to form the transverse plane (XY) of the face perpendicular to the midfacial plane (YZ) (Figure 8). The bilateral exocanthions serve as the points of origin, establishing the positive and negative directions for both XY axes. A line extending from the origin toward the chin, parallel to the midfacial (sagittal) plane (YZ) and perpendicular to the XY-axis, determines the negative direction of the YZ-axis (Figure 9: Red vector). For the XY-axis, a line extending from the origin to the left side of the face, parallel to the XY-axis and perpendicular to the midfacial (sagittal) plane (YZ), is used to identify the negative direction of the XY-axis (Figure 9: Green vector). The positive directions of the YZ and XY axes are determined by projecting in the opposite direction from the new origin. The cross-product of the new YZ and XY axes is used to establish the coronal plane (XZ) (Figure 9: Blue vector).

##### Landmark Identification

Deep MVLM was used for landmark identification. Using this model, the 3D point cloud model of each image was rendered from multiple views, and for each view, landmark candidates were estimated. The facial images were rendered using RGB (red, green, blue color) texture, geometry, curvature, and depth. The model was pre-trained and validated for different render combinations based on the BU-3DFE and UPM-3DFE datasets. The *x*, *y*, and *z* coordinates of the seven bilateral facial landmarks of interest (Table 1) were identified and then exported to a Microsoft Excel spreadsheet.

### 2.4. Evaluation of Facial Asymmetry

Facial asymmetry was evaluated in each 3D facial image using the coordinates of the landmarks identified manually and through the deep MVLM-based method. Since only the amount of asymmetry was of interest, not its direction, the absolute differences in coordinates between the right and left sides were used to calculate the asymmetry index for each of the seven bilateral facial landmarks, representing the total amount of asymmetry for these points. The following formula was employed: √((*X_l_ − X_r_*)^2^ + (*Y_l_ − Y_r_*)^2^ + (*Z_l_ − Z_r_*)^2^), where *x*, *y*, and *z* denote the coordinates of a landmark, the subscript *l* represents the left side, and *r* represents the right side [37]. The asymmetry index formula was initially introduced by Katsumata et al. for assessing facial asymmetry in CT images [8]. The asymmetry indices of the landmarks identified manually were compared to those identified using the deep MVLM-based method. All asymmetry indices were expressed as linear distances in mm.

### 2.5. Statistical Analysis

To assess intra-rater reliability, Observer 1 re-identified landmarks and calculated the asymmetry indices for 20 images at 2-week intervals [42]. Inter-rater reliability between the two observers was evaluated when each observer independently identified landmarks for all the subjects, and asymmetry indices derived from the *x*, *y,* and *z* coordinates of the seven bilateral facial landmarks were calculated. The degree of agreement between asymmetry indices was assessed for intra and inter-rater reliability using intraclass correlation coefficient (ICC). The procedure was a two-way mixed model using a consistency definition with 95% confidence intervals (CI). ICC values less than 0.5 indicated poor reliability, values between 0.5 and 0.75 indicated moderate reliability, values between 0.75 and 0.9 indicated good reliability, and values greater than 0.90 indicated excellent reliability [43]. The ICC was conducted with the SPSS software program, version 29.0 for Macintosh (IBM Corp., Armonk, NY, USA).

To validate the automated artificial intelligence-based method in landmark identification and facial asymmetry evaluation, the asymmetry indices obtained by this method were compared to those obtained from the manual method by observer MA. The Shapiro–Wilk test was used to assess the normality of the data and indicated that the data was not normally distributed. Therefore, the Wilcoxon signed-rank test was used to compare the medians of the asymmetry indices of the landmarks obtained by each method. The normality assessment and the Wilcoxon signed-rank test were conducted using JMP^®^, Version <17>. SAS Institute Inc., Cary, NC, USA. *p*-values less than 0.05 were considered statistically significant. Correction for multiple testing using the Benjamini–Hochberg procedure (False Discovery Rate equals 0.05) was performed to decrease the type I error [44]. 

## 3. Results

The summarized results of both intra- and inter-rater reliability assessments, including the corresponding 95% CI, to evaluate the reproducibility of landmark selection and associated index measurements are presented in Table 2. For intrarater reliability, the ICC values ranged from 0.62 (indicating moderate agreement) to 0.99 (indicating excellent agreement) across the seven bilateral facial landmarks. On average, the intrarater ICC was calculated to be 0.76, suggesting a good overall agreement between repeated measurements by the same rater [43]. These results indicate that the asymmetry indices based on the 3D coordinates of the landmarks were generally consistent when measured by the same rater. Regarding interrater reliability, the ICC values varied between 0.72 (moderate agreement) and 0.96 (excellent agreement) across the same set of landmarks. The average interrater ICC was determined to be 0.82, signifying a good overall agreement between different raters in measuring the asymmetry indices [43]. These results indicate that the method used to derive asymmetry indices from the 3D coordinates demonstrated reliability not only within the same rater but also when assessed by different raters.

The asymmetry indices for the seven bilateral landmarks, identified through manual and automated methods, are summarized in Table 3. In support of the null hypothesis, results from the Wilcoxon signed-rank test indicated no statistically significant difference between the manual and automated techniques for calculating asymmetry indices in the case of palpebrale superius, palpebrale inferius, exocanthion, endocanthion, and crista philtra. However, statistically significant differences were observed for the asymmetry indices of alare (*p* = 0.0056) and cheilion (*p* = 0.0081) between the manual and automated methods (Table 3). To further evaluate agreement between the two methods, Bland–Altman analysis was performed (Supplemental Figure S1; Supplemental Table S1).

## 4. Discussion

In this study, we aimed to validate an artificial intelligence-based software for assessing facial asymmetry by comparing it with a manual method. First, we sampled N = 130 patients and generated 3D surface facial images from the Vectra^®^ M3 stereophotogrammetry imaging system. Then, observer MA manually selected seven bilateral facial landmarks and calculated their asymmetry indices as the square root of the sum of the squared differences between each bilateral *x*, *y*, and *z* landmark coordinate. Subsequently, we applied a deep multi-view learning model [32] trained on the 3D surface facial images of N = 150 individuals to automatically identify landmarks on N = 130 patients in our study. Finally, we compared the asymmetry indices from the manual method to those derived from the deep MVLM.

The intrarater reliability for manual measurements taken at a 2-week interval ranged from moderate to excellent (0.62 to 0.99). Similarly, the interrater reliability for manual measurements demonstrated moderate to excellent consistency (0.72 to 0.96). Our findings demonstrate slightly lower reliability for a couple of landmarks. This could be attributed to conducting the ICC analysis on the asymmetry indices rather than the direct linear measurements. The computation of asymmetry indices that apply a coordinate-based formula, as conducted in our study, tends to magnify small variations in landmark positioning [30]. In clinical practice, the proportions and the indices are more important than the absolute values of distances [30,45].

The results of the Wilcoxon signed-rank test revealed a comparable precision of the artificial intelligence-based software compared to the manual method in calculating the asymmetry indices. There was no statistically significant difference between the manual and automated techniques for calculating asymmetry indices in the case of palpebrale superius, palpebrale inferius, exocanthion, endocanthion, and crista philtra. However, statistically significant differences were observed between the manual and automated methods for the asymmetry indices of alare (*p* = 0.0056) and cheilion (*p* = 0.0081). Factors underlying our results are associated with the study design and include (i) the choice of a reference plane relative to the facial anatomy, (ii) the features of our training dataset for applying the deep MVLM, (iii) and the nature of landmark sampling on 3D surface images.

In this study, the midfacial plane was determined as the plane passing through the nasion and being perpendicular to both bilateral exocanthions [37]. A previous report evaluated 90 3D facial images to compare four reference planes perpendicular to the bilateral exocanthion, endocanthion, superalare, and cheilion. Their conclusion indicated that the most effective reference plane for evaluating 3D facial asymmetry is the one perpendicular to and dividing the line connecting the bilateral exocanthions [46]. Nevertheless, the midpoint of the line between the bilateral exocanthions might not align with the position of any of the midfacial landmarks in the x coordinate [37]. For clinical purposes, several studies have suggested that opting for the midfacial plane, which passes through the nasion and is perpendicular to the plane connecting both exocanthions and the nasion, is preferable [47,48]. Since soft tissue nasion typically resides within a depression between the eyes and slightly above the nasal bridge, its reproducibility commonly surpasses that of other anatomical facial landmarks [37]. 

In this study, the automated analysis conducted landmark identification using the deep MVLM, which utilized a pre-trained artificial intelligence model trained on data obtained from two publicly available datasets: BU-3DFE and UPM-3DFE [32]. The training process for the BU-3DFE dataset involved rendering faces utilizing various combinations of RGB texture, geometry, curvature, and depth rendering techniques. Conversely, the UPM-3DFE dataset training exclusively utilized RGB rendering [32]. RGB texture rendering captures an object or facial surface appearance using red, green, and blue color data. In the context of facial landmark identification, it specifically portrays the facial surface using color and texture details, encompassing skin tone, texture, shading, and related color features [49]. Geometry rendering acquires the geometric details of the facial surface, including contours, shapes, and spatial arrangement. Integrating geometry rendering into the landmark identification model enables algorithms to comprehend the structural aspects of the face, facilitating landmark localization through analysis of the facial structure in its three-dimensional coordinates [32,50,51]. Expanding on geometric rendering, curvature rendering focuses on analyzing local surface changes. It involves calculating metrics like mean or Gaussian curvature at various facial points, aiding in differentiating between flat and curved regions. This helps to precisely locate landmarks associated with specific curvature patterns on the face [32,51]. Depth rendering provides data on the distance between facial surface points and a reference plane, clarifying relative depths among landmarks. This information aids algorithms in evaluating facial feature arrangement, improving accurate landmark identification, especially regarding their z-axis (depth) placement [32,51]. Integrating these rendering techniques enriches the dataset used to train facial landmark identification models. This comprehensive data captures not only color and texture details but also geometric structure, curvature variations, and depth information, which enables algorithms to have a more holistic understanding of facial surfaces. Consequently, it enhances the accuracy and robustness of facial landmark identification systems by incorporating multidimensional data for analysis and recognition of key facial landmarks [32]. 

The results of this study revealed discrepancies in computing the asymmetry indices of the alare and cheilion landmarks between the manual method and the artificial intelligence-based software. This discrepancy suggests that the artificial intelligence-based software could not identify the paired alare and cheilion landmarks with human accuracy. Identifying paired landmarks is generally more challenging for humans than midline landmarks [11]. The landmarks that are typically challenging for humans to locate often exhibit high errors when identified using artificial intelligence-based software [32]. Previous studies on the reproducibility of landmark identification in 3D facial images revealed lower reproducibility in bilateral nose landmarks, such as the paired alare and paired alar curvature [11]. Regarding nose-related landmarks, their vertical positioning demonstrates greater consistency; however, in other spatial planes, the reproducibility of these landmarks appears to be lower. This tendency might be related to the contour of the nose in this area. For instance, a landmark placed on the convexity of the nose will have a different *z* coordinate to one placed in the alar fold. This clearly relays on the rater’s perception of the landmark definition [52]. Moreover, unlike 2D images, using 3D facial images in manual landmark identification provides multiple views for visualization, potentially influencing the rater’s judgment on landmark placement and allowing for more precise placement. Another report concluded that the variation among the various landmarks on the face can be attributed to a good description/definition of each landmark [12]. A previous report proposed new definitions of facial landmarks based on surface curvature, offering a genuinely three-dimensional approach. The results demonstrated reduced variation among observers in landmark identification when utilizing these new 3D definitions [53]. The distinctive features of the landmarks can also impact landmark identification [12]. For example, a point-associated landmark such as crista philtra is prone to fewer errors than one on a flat surface like the alare [12]. Cheilion is defined as the point located at each labial commissure [11]. The positioning and shape of lip commissures vary depending on factors such as lip morphology, the tone of perioral muscles, the position of incisors, and the morphology and positioning of the mandible [54,55]. The identification of the cheilion is sometimes challenging due to its location in a sharp transitional area between the vermilion border and the surrounding skin, which may not always be distinctly recognizable [56]. For example, in certain individuals, due to the potential influence of aforementioned characteristics, the lip commissure becomes an area rather than a defined point. This, in turn, will impact the rater’s assessment of the cheilion landmark. On the other hand, artificial intelligence-based software may rely primarily on changes in skin color (i.e., RGB on the 3D facial image) or texture in this area to identify the transitional area, without being affected by the diverse factors influencing the shape and positioning of the lip commissure that can impact a human rater’s assessment of the location of cheilion [32]. Despite the reported inconsistency between the two methods in calculating the asymmetry indices of two landmarks, the observed asymmetry values did not exceed thresholds considered clinically significant for treatment planning (3 mm) [33]. The largest observed median asymmetry index was 2.77 mm (Cheilion, manual method), which remained within the clinically acceptable limit.

Finally, we note that the evaluation of facial asymmetry on 3D images can be carried out using either a landmark-based or surface-based method. Landmark-based methods involve manually identifying landmarks on 3D facial images to obtain a range of 3D measurements for identifying facial asymmetry. On the other hand, surface-based methods are landmark-independent approaches that consider thousands of points of the facial surface and allow a full-face analysis. Alqattan et al. compared the diagnostic abilities of the landmark-based and the surface-based methods of 3D analysis of facial asymmetry [57]. They revealed that both analyses accurately support the diagnosis of facial asymmetry. The surface-based analysis has the advantage of using several thousand cloud points captured by the scanning equipment. Nevertheless, the cloud point configuration may be insufficient in detecting clinically relevant asymmetry in a particular facial region. One advantage of the landmark-based method over surface-based techniques is its increased capability in detecting clinically relevant asymmetry within specific facial regions [57]. Consequently, this method allows for identifying optimal surgical landmark relocation to achieve symmetrical repositioning, particularly when surgical correction is indicated [8]. However, the landmark-based method possesses the disadvantages of being time-consuming, and the inter-examiner’s variability for some landmarks can be high [57]. In our investigation, we utilized artificial intelligence-based software to mitigate these disadvantages.

To our knowledge, our study is the first to validate artificial intelligence-based software and a 3D landmark-based method to identify patients with facial asymmetry. Automated software significantly reduces the time required for facial asymmetry assessment. The automated system is markedly more efficient, with the computation of the asymmetry index taking approximately 10–12 min for each case through manual assessment. In contrast, the automated process accomplished the same task for the whole sample in mere seconds, underscoring its remarkable efficiency and time-saving capabilities. In addition, it minimizes the potential for high inter-examiner variability, consequently enhancing precision and efficiency. However, the results showed that the software could not accurately calculate the asymmetry index of the alare and cheilion landmarks based on their *x, y,* and *z* coordinates. While the software’s deep MVLM is notable for its unique feature of rendering 3D images from multiple viewpoints, further investigation is necessary to evaluate the model’s proficiency by enlarging the training dataset and testing different landmark configurations. This investigation might provide crucial insights to enhance the model training, accounting for factors that could alter the morphology of the facial soft tissue, particularly in regions sampling landmarks, for example. Moreover, re-landmarking the training dataset by incorporating the updated 3D curvature-based definition of facial landmarks could augment the system’s accuracy. For improved accuracy, the software should be able to factor in the dynamic changes in soft tissue morphology, ensuring it accurately identifies landmarks amidst such variations. Future improvements could focus on conducting regional, rather than solely point-based, analyses to better capture asymmetry patterns and enhance clinical relevance. Moreover, future investigations should evaluate the model on patients with more severe facial asymmetry and perform external validation across different clinical centers and populations beyond orthodontic patients to assess generalizability and potential domain shift.

## 5. Conclusions

- The automated method proves notably more efficient than the manual technique for evaluating facial asymmetry using 3D facial images.
- The artificial intelligence-based software exhibits comparable reliability to the manual approach when calculating the asymmetry index based on 3D landmark coordinates.
- The disagreement observed between the automated and manual methods in a couple of the facial landmarks can be addressed through further improvement of the automated software. This may entail additional training of the software, considering the dynamic nature of soft tissues, and integrating updated 3D definitions of facial landmarks into the dataset.
- This automated technique is valuable for orthodontic practitioners and researchers, fostering progress toward an evidence-based practice with enhanced efficiency.
- Additionally, this method’s versatility suggests its potential extension for evaluating other facial features beyond asymmetry assessment.

## Figures and Tables

**Table 1:** Definitions of 7 bilateral facial soft tissue landmarks.

Landmark *	Definition
Palpebrale superius ^6,7^	Superior mid-portion of the free margin of upper eyelids
Palpebrale inferius ^8,9^	Inferior mid-portion of the free margin of lower eyelids
Exocanthion ^1,3^	The soft tissue point located at the outer commissure of each eye fissure
Endocanthion ^4,5^	The soft tissue point located at the inner commissure of each eye fissure
Alare ^10,11^	The most lateral point on each alar contour
Crista philtra ^12,13^	The point at each crossing of the vermilion line and the elevated margin of the philtrum
Cheilion ^14,15^	The point located at each labial commissure

* Superscript refers to numbers in Figure 3.

**Table 2:** Summary of results from ICC.

	Intrarater ICC (Main Observer)	95% CI	Interrater ICC	95% CI
Palpebrale superius	0.991	0.976–0.996	0.756	0.651–0.830
Palpebrale inferius	0.62	0.410–0.850	0.851	0.786–0.896
Exocanthion	0.607	0.008–0.845	0.719	0.598–0.804
Endocanthion	0.716	0.283–0.888	0.944	0.920–0.961
Alare	0.778	0.440–0.912	0.657	0.509–0.760
Crista philtra	0.624	0.051–0.851	0.855	0.793–0.899
Cheilion	0.963	0.906–0.985	0.959	0.942–0.972

**Table 3:** Summary of the descriptive statistics of the asymmetry indices ^1^ obtained by the manual and the automated deep MVLM methods and the comparison of the medians of each method using Wilcoxon signed-rank test.

		Manual Method					Automated Deep MVLM Method							Wilcoxon Signed-Rank Test *p*-Values
Landmarks	Median	25th Percentile (Q_1_)	75th Percentile (Q_3_)	Mean	SD	Median	25th Percentile (Q_1_)	75th Percentile (Q_3_)	Mean	SD	Median Paired Difference	Hodges–Lehmann 95% CIs	*p*-Value	*p*-Value Adjusted * and Benjamini–Hochberg FDR Significance
Palpebrale superius	2.65	1.75	3.79	2.85	1.48	2.51	1.64	3.48	3.24	5.96	0.11	−3.81–3.87	0.4618	0.461 NS
Palpebrale inferius	2.46	1.64	3.58	2.68	1.27	2.13	1.51	3.40	2.48	1.43	0.31	−4.13–3.35	0.0565	0.132 NS
Exocanthion	2.69	1.69	4.05	3.03	1.77	2.67	1.85	4.24	3.20	1.92	−0.14	−4.65–4.64	0.4064	0.462 NS
Endocanthion	1.89	1.46	2.62	2.14	1.06	1.7	1.18	2.47	2.25	3.74	0.16	−2.75–2.78	0.1223	0.214 NS
Alare	2.05	1.31	2.74	2.15	1.11	1.54	1.09	2.09	1.70	0.96	0.39	−1.64–3.21	0.0008	**0.0056 SIG**
Crista philtra	1.42	0.87	2.54	1.91	1.56	1.33	0.85	2.04	1.62	1.26	0.18	−3.37–4.38	0.2004	0.281 NS
Cheilion	2.77	2.00	3.80	3.15	1.72	2.30	1.57	3.31	2.56	1.38	0.54	−3.55–5.15	0.0023	**0.0081 SIG**

^1^ Asymmetry index formula: √((*X_l_ − X_r_*)^2^ + (*Y_l_ − Y_r_*)^2^ + (*Z_l_ − Z_r_*)^2^), where *x*, *y*, and *z* denote the coordinates of a bilateral landmark, the subscript *l* represents the left side, and *r* represents the right side. * *p*-Value adjusted using Benjamini–Hochberg procedure to determine “*p*” values (False Discovery Rate FDR = 0.05), and statistical significance; NS = Not Significant, SIG = Significant [44]. All units are in mm.

## Data Availability

The data presented in this study are available from the corresponding author upon request.

## Supplementary Materials

The following supporting information can be downloaded at: https://www.mdpi.com/article/10.3390/jcm14207172/s1, Figure S1: Bland–Altman plots comparing manual and automated asymmetry index measurements across facial landmarks; Table S1: Bland–Altman analysis of agreement between manual and AI-derived asymmetry indices.

## Abbreviations

The following abbreviations are used in this manuscript:

AI	Artificial intelligence
ICC	Intraclass correlation
2D	Two-dimensional
PA	Posteroanterior
CT	Computed tomography
CBCT	Cone beam computed tomography
MVLM	Deep multi-view learning model
VAM	VECTRA^®^ 3D Analysis Module
MM	Millimeters
CLAIM	Checklist for Artificial Intelligence in Medical Imaging
BU-3DFE	Binghamton University 3D Facial Expression dataset
UPM-3DFE	Universiti Putra Malaysia Facial Expression Recognition Database
RGB	Red, Green, Blue color
CI	Confidence intervals
