# Supplementary material for: Precision Assessment of Facial Asymmetry Using 3D Imaging and Artificial Intelligence
Source: J Clin Med. 2025 Oct 11;14(20):7172. doi: 10.3390/jcm14207172 (PMC12565447; doi:10.3390/jcm14207172)
Supplement: Supplementary file 1 [file jcm-14-07172-s001.zip › Supplemental figure 1- Table 1.pdf]

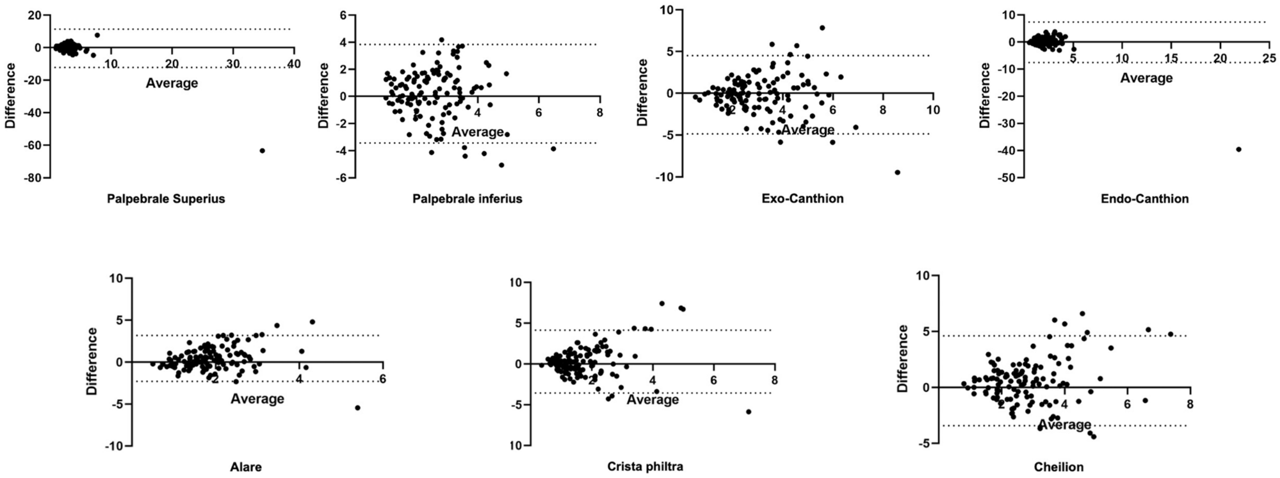

Supplemental Figure S1: Bland–Altman plots comparing manual and automated asymmetry index measurements across facial landmarks. Most landmarks showed good agreement, while **Alare** and **Cheilion** exhibited the largest variability and least consistency (*P* = 0.0056 and *P* = 0.0081).

Supplemental Table S1: Bland–Altman analysis of agreement between manual and AI-derived asymmetry indices.

	Bias	95% Limits of Agreement
Palpebrale superius	-0.39	-12.2 – 11.40
Palpebrale inferius	0.20	-3.43 – 3.83
Exocanthion	-0.17	-4.85 – 4.50
Endocanthion	-0.12	-7.58 – 7.36
Alare	0.44	-2.28 – 3.18
Crista philtra	0.28	-3.56 – 4.14
Cheilion	0.59	3.42 – 4.60

All units are in mm.
